# The Double-Edged Sword Effect of the Fibrinolytic System in Alzheimer’s Disease

**DOI:** 10.1007/s10571-026-01699-6

**Published:** 2026-02-27

**Authors:** Mingqing Tang, Meimei Liang, Xianying Zhang, Chunzhan Hong, Lichao Ye

**Affiliations:** 1 Medical School, Huaqiao University, Quanzhou, 362021 Fujian China; 2 Neurology Department, The 2 nd Affiliated Hospital of Fujian Medical University, Quanzhou, 362021 Fujian China

**Keywords:** Alzheimer's disease, Amyloid-β, Fibrinolytic system, Plasmin, Plasminogen activator

## Abstract

Alzheimer’s disease (AD), the most prevalent neurodegenerative disorder, is fundamentally driven by dysregulation between amyloid-β (Aβ) production and clearance. Although multiple Aβ clearance pathways have been reported, the fibrinolytic system remains the only enzymatically validated degradation route. Notably, a striking paradox is emerging: fibrinolytic agents exhibit both neuroprotective and AD-promoting effects in clinical observations. This duality necessitates urgent investigation into the fibrinolytic system’s double-edged sword mechanism in AD pathogenesis. This review provides the first comprehensive analysis of the fibrinolytic system’s dichotomous regulatory functions in AD progression, summarizes current advancements in the pathogenesis and therapeutic interventions, and proposes novel research directions. By resolving this molecular paradox, we aim to accelerate transformative breakthroughs in AD prevention and precision medicine strategies. Fibrinolytic System: Neuroprotection vs. Pathogenesis. The fibrinolytic system alleviates AD by directly degrading Aβ and indirectly improving the microenvironment of the central nervous system and cerebrovascular system. Meanwhile, this system also accelerates AD by promoting neuroinflammation and tau hyperphosphorylation.

**Graphical Abstract:**

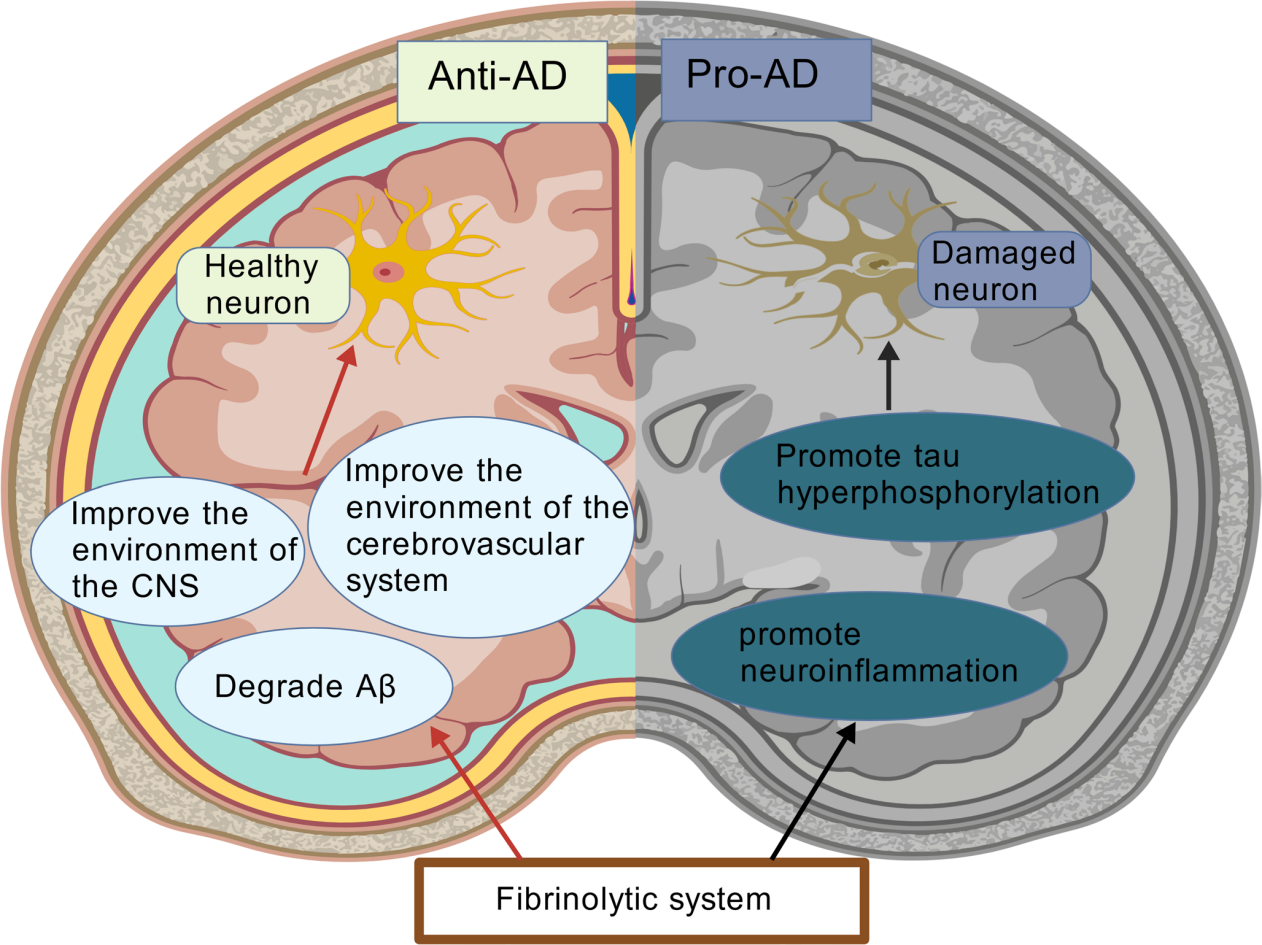

**Supplementary Information:**

The online version contains supplementary material available at 10.1007/s10571-026-01699-6.

## Introduction

AD, the most prevalent neurodegenerative disorder, predominantly affects the elderly and demonstrates familial inheritance patterns. It is clinically characterized by progressive dementia, which includes memory decline, language impairment, behavioral disturbances, and neuronal degeneration. Regrettably, AD remains incurable to date (Soni et al. 2025). AD progresses through three pathologically distinct phases. The initial ‌asymptomatic phase‌ (10–20 years pre-symptomatic) features abnormal Aβ deposition due to the replacement of physiological cleavage (α-secretase) of amyloid beta precursor protein (APP) by pathological cleavage (β/γ-secretase), generating neurotoxic Aβ oligomers and extracellular senile plaques that induce synaptic dysfunction, neurotransmitter interference (Wang et al. 2014a, b), chronic neuroinflammation (Forloni and Balducci 2018), and early tau hyperphosphorylation. The subsequent ‌prodromal phase‌ manifests as mild cognitive impairment due to progressive synaptic loss, particularly in hippocampal circuits, and degeneration of cholinergic neurons in the basal forebrain region, which is highly vulnerable to Aβ and tau toxicity (Oliveira et al. 2017). This cholinergic deficit exacerbates cognitive decline by reducing acetylcholine synthesis, while disrupted calcium homeostasis further potentiates synaptic dysfunction (Do Carmo et al. 2021). The terminal ‌dementia phase‌ involves widespread neurodegeneration driven by intracellular Aβ oligomers, which hyperphosphorylate tau protein, forming neurofibrillary tangles (NFTs) that destabilize neuronal cytoskeletons (Ng et al. 2019).

It is widely acknowledged that the imbalance between Aβ production and clearance is just the driving force of AD, persisting throughout all three stages (de Oliveira et al. 2021). Currently, four key clearance mechanisms have been reported to maintain Aβ homeostasis: (1) Proteolytic degradation, mainly mediated by the fibrinolytic system, which directly degrades Aβ into non-toxic fragments (Tucker et al. 2000); (2) Transvascular clearance mediated by LRP-1 across the blood-brain barrier (BBB) (Akhter et al. 2018); (3) Glymphatic drainage via the glymphatic system (Ben-Nejma et al. 2023); (4) Peripheral uptake enhanced by agents such as polysaccharide Krestin (PSK) (Chen et al. 2022). Among these, the fibrinolytic pathway is particularly promising due to its direct Aβ degradation capability. In addition to its potent direct Aβ degradation capability (Jacobsen et al. 2008), the fibrinolytic system also maintains neuroprotective functions under physiological conditions by cleaving brain-derived neurotrophic factor (Pro-BDNF) into mature brain-derived neurotrophic factor (mBDNF) (Gray and Ellis 2008). These findings increasingly heighten our anticipation that the fibrinolytic system will exert broader, more extensive, and stronger protective effects in AD. Notably, a novel facultative plasminogen-independent fibrinolytic agent (snFPITE) was first reported by our team (Tang et al. 2023, 2025; Lin et al. 2023; Kang et al. 2024). Further in vivo experiments indicated that AD mice might benefit from long-term administration of snFPITE. However, a recent report presented a paradoxical result, indicating that the fibrinolytic system may exacerbate neuroinflammation and accelerate neurodegeneration in pathological states (Baker et al. 2019).

This dual role of the fibrinolytic system in AD pathophysiology urgently requires further investigation. However, no comprehensive review has been published on such duality. Although most existing studies have centered on the protective role of the fibrinolytic system via the direct degradation of insoluble Aβ plaques (Esser et al. 2024; Yepes 2021), this review is the first to systematically elaborate on the double-edged sword property of this system. i.e., besides abrogating Aβ toxicity, fibrinolytic system activation may exacerbate neurovascular unit damage through mechanisms such as BBB disruption and fibrinogen extravasation. Moreover, the traditional view holds that fibrinolytic dysfunction contributes to AD pathogenesis only by modulating Aβ metabolism (Huang et al. 2025; Zhang et al. 2021). Conversely, we integrate the latest evidences to reveal its intimate association with tau pathology and synaptotoxicity (Simoes-Pires et al. 2025). Therefore, this work presents the first comprehensive analysis of the fibrinolytic system’s dual regulatory functions in AD progression, along with a summary of the latest advances in AD pathogenesis pathways and therapeutic strategies. By elucidating this molecular paradox, we aim to promote the transformative advances in AD prevention and precision medicine.

## Pathogenesis of AD

The scientific understanding of AD’s pathogenesis has undergone profound evolution since its initial pathological characterization in 1906 by German physician Alois Alzheimer, who first documented the distinctive amyloid plaques and NFTs that would later define the disorder (Salloway 2008). This seminal discovery established the morphological foundation for subsequent investigations, which progressively unraveled the molecular mechanisms underlying AD pathogenesis. The late 20th century marked critical breakthroughs, including the identification of Aβ as the principal component of senile plaques (Wisniewski and Wrzolek 1988), followed by the elucidation of tau protein pathology and its role in NFT formation (Medina and Avila 2014). As research progressed into the 21 st century, the pathogenic framework expanded to encompass multiple interconnected mechanisms, notably Aβ aggregation (the central driver), tau hyperphosphorylation, cholinergic system degeneration, and calcium homeostasis disruption (Daly et al. 2020). This evolutionary trajectory reflects the field’s paradigm shift from descriptive neuropathology to mechanistic molecular biology, while consistently prioritizing Aβ pathology as the predominant therapeutic target throughout the research continuum.

These multifaceted pathogenic mechanisms underscore the inherent complexity of AD pathogenesis, which is marked by intricate cascade reactions and interactions among multiple pathways, rather than being attributed to singular causative factors. Therefore, future research should adopt a systems biology approach to elucidate the dynamic interplay among intercellular communication, epigenetic regulation, and metabolic networks, and ultimately unravel the complexity of AD.

### Aβ aggregation-Induced AD

**Fig. 1:**
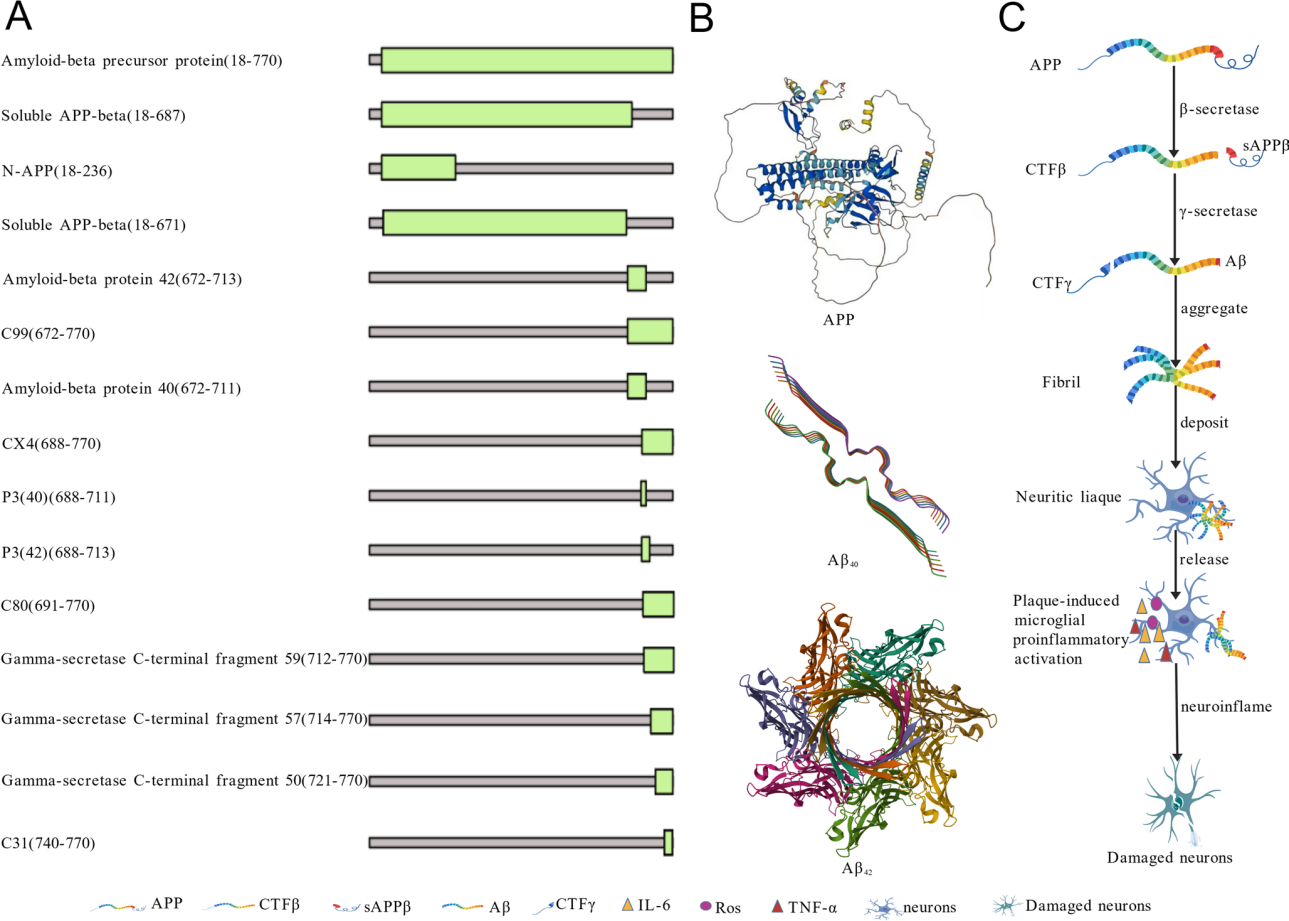
Aβ aggregation-induced AD. **A** Representative fragments of processed APP; **B** Representative structures of processed APP; **C** The pathway of the Aβ aggregation-driven AD

Compelling evidence has established Aβ aggregation as the central driver of AD pathogenesis (Huang et al. 2025; Price and Sisodia 1998). This pathological process stems from altered cleavage of APP (P05067, 770 amino acids, 86943 Da), a cell surface receptor that performs physiological functions on the surface of neurons relevant to neurite growth, neuronal adhesion, and axonogenesis (Jumper et al. 2021; Fleming et al. 2025). Under normal physiological conditions, APP will be cleaved by α-secretase, generating neuroprotective alpha-cleaved soluble APP (sAPPα) and a corresponding cell-associated C-terminal fragment (C83). The latter is further processed into non-aggregatable, non-pathogenic smaller fragments, such as the P3 fragment. However, once APP is altered and cleaved by pathological β-secretase (BACE1) at the N-terminus of the Aβ peptide sequence, between residues 671 and 672, this leads to the generation and extracellular release of beta-cleaved soluble APP (sAPPβ), and a corresponding cell-associated C-terminal fragment (CTFβ, C99), which is subsequently cleaved by γ-secretase, generating β-sheet-rich Aβ peptides and a corresponding cell-associated C-terminal fragment (CTFγ) (Lin et al. 2000; Miranda et al. 2021; Okada et al. 2010). Interestingly, the γ-secretase cleaves APP at variable sites within the transmembrane domain, generating Aβ peptides ranging from 36 to 43 residues in length (Selkoe and Wolfe 2007) (Fig. 1A). Among the resultant isoforms, Aβ1–42 exhibits a particularly strong propensity for self-aggregation compared to Aβ1–40 and other shorter peptides (Fig. 1B). Therefore, an elevated Aβ42/Aβ40 ratio has become a common biochemical feature in early-onset familial AD (FAD) (Yang et al. 2020).

Subsequently, the neurotoxic cascade is triggered when soluble Aβ monomers spontaneously assemble into oligomers and fibrils, ultimately depositing as extracellular plaques. These aggregates trigger multifaceted pathological consequences: (1) Clearance dysfunction. Failed microglial phagocytosis of Aβ leads to persistent release of pro-inflammatory mediators (ROS, TNF-α, IL-1β), establishing chronic neuroinflammation that directly damages synapses and recruits additional immune cells (de Oliveira et al. 2021); (2) Vascular compromise. Sustained inflammation disrupts blood-brain barrier integrity, permits fibrinogen infiltration while impairing metabolic waste clearance, and creates a feed-forward loop that exacerbates both Aβ deposition and neuroinflammation (de Oliveira et al. 2021); (3) Mitochondrial dysfunction. Aβ accumulation within mitochondria specifically inhibits complexes II/IV of the electron transport chain, reducing ATP synthesis while increasing ROS production. This oxidative stress further promotes amyloidogenesis by activating β-secretase (Onyango et al. 2021); (4) Immune activation. Aβ binding to the CD14 receptor initiates innate immune responses, recruiting activated microglia and astrocytes around plaques. This establishes localized chronic inflammation, which amplifies neuronal damage (Onyango et al. 2021). These interconnected pathways collectively establish a self-perpetuating cycle in which Aβ aggregation both initiates and amplifies neurodegenerative processes, ultimately driving AD progression through synergistic mechanisms (Fig. 1C).

### Acetylcholine Deficiency Induced AD

**Fig. 2:**
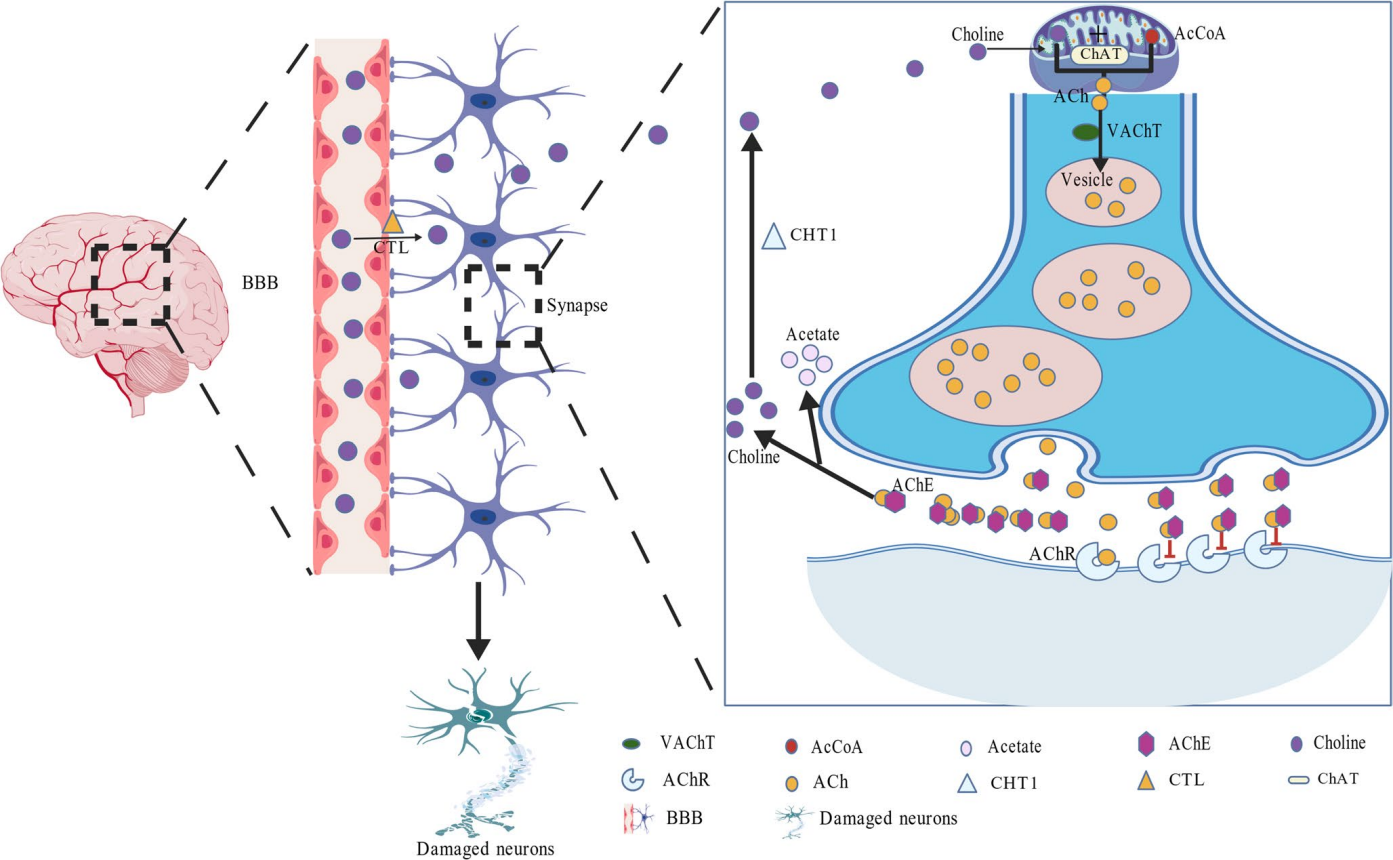
ACh deficiency induced AD

It is widely acknowledged that acetylcholine (ACh) is an essential neurotransmitter for proper central nervous system function (Grünewald and Siefert 2019; Onyango et al. 2021). The availability of ACh is tightly regulated by multiple distinct molecular events, including the transport and recycling of choline, as well as the synthesis and degradation of Ach; any disturbance of these stages will lead to ACh deficiency, causing cholinergic signaling deficits and degeneration of cholinergic neurons (Fig. 2). Generally, circulating choline needs to cross the BBB effectively through choline transporter-like proteins (CTL-1 and CTL-2) to maintain appropriate levels of neuronal choline concentrations. Then, the biosynthesis of ACh in neurons is initiated by the mitochondrial enzyme choline acetyltransferase (ChAT), which catalyzes the conversion of choline and acetyl-CoA into ACh. Therefore, impaired ChAT activity can lead to impaired ACh synthesis. Once synthesized, ACh is packaged into vesicles via ACh transporters (AChT) and released into the synaptic cleft upon neuronal depolarization. Notably, elevated acetylcholinesterase (AChE) levels accelerate neurotransmitter degradation, resulting in substantially diminished synaptic ACh concentrations. Under normal physiological conditions, choline is recycled back into presynaptic neurons through the high-affinity choline transporter 1 (CHT1) for ACh resynthesis. In AD pathogenesis, disrupted CHT1 expression and function create a choline-deficient state that becomes the rate-limiting factor in ACh production.

Neuropathological investigations have identified a characteristic degeneration of cholinergic neurons in the basal forebrain of patients with AD, marked by significant neuronal loss and substantial impairment of axonal projections to cognitively relevant regions, including the hippocampus and cerebral cortex. This dual pathology severely disrupts cholinergic neurotransmission in key brain areas, forming the neurobiological foundation of the cholinergic hypothesis (Talita et al. 2016). Therefore, impaired CTL-mediated choline transport, disrupted ChAT-mediated ChA biosynthesis, accelerated AChE-dependent ChA degradation, and compromised CHT1-mediated choline recycling lead to ACh deficiency and subsequent AD pathogenesis. Similarly, dysfunction or reduced activity of postsynaptic acetylcholine receptors (AChRs) may exacerbate cholinergic signaling deficits due to impaired neurotransmitter reception.

### Tau Protein Hyperphosphorylation Induced AD

**Fig. 3:**
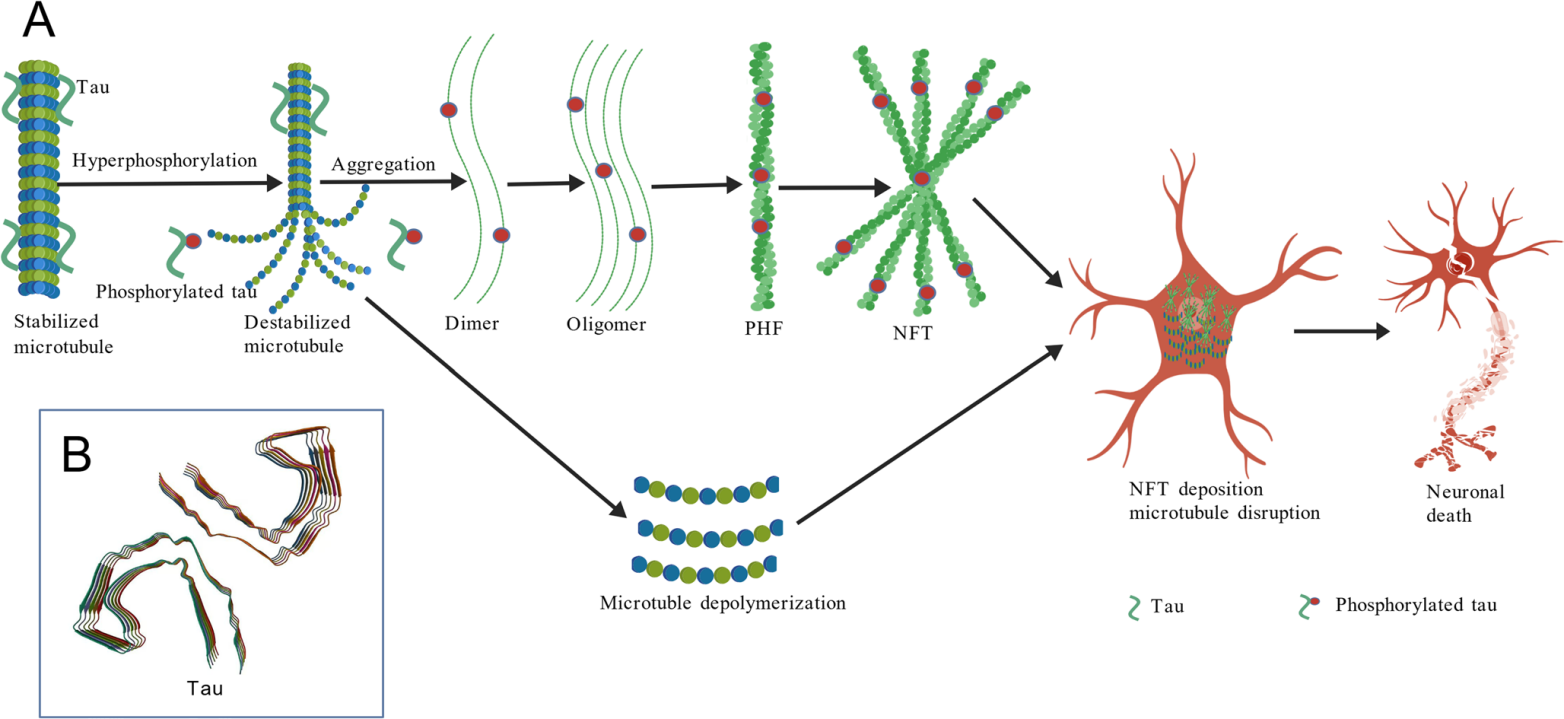
Tau protein hyperphosphorylation induced AD. **A** Tau hyperphosphorylation-mediated AD pathogenesis; **B** Structure of tau protein

An alternative perspective on AD pathogenesis focuses on tau protein hyperphosphorylation (Fig. 3A). As a microtubule-associated protein (Fig. 3B), tau normally exists in an unfolded, thermostable state that promotes microtubule assembly and stability, and might be involved in the establishment and maintenance of neuronal polarity (Yoshida and Goedert 2012). The C-terminus binds axonal microtubules while the N-terminus binds neural plasma membrane components, suggesting that tau functions as a linker protein between the two (Yoshida and Goedert 2012; Sandberg et al. 2020). Tau is phosphorylated at serine and threonine residues in S-P or T-P motifs by proline-directed protein kinases (PDPK1, CDK1, CDK5, GSK3, MAPK) (only 2–3 sites per protein in interphase, a seven-fold increase in mitosis, and in the form associated with paired helical filaments (PHF-tau), and at serine residues in K-X-G-S motifs by MAP/microtubule affinity-regulating kinase (MARK1, MARK2, MARK3, or MARK4). This phosphorylation causes detachment from microtubules, and subsequent microtubule disassembly (Gu et al. 2013; Drewes et al. 1995). Therefore, pathological hyperphosphorylation of tau protein triggers microtubule destabilization, leading to phosphorylated tau accumulation and dimer formation. These dimers progressively aggregate into oligomeric species, ultimately developing into the characteristic filamentous structures, NFTs, the defining neuropathological hallmark of AD (Man et al. 2023).

Moreover, NFT distribution follows a stereotypical spatiotemporal progression in AD pathogenesis, initially localizing to medial temporal lobe structures (including the entorhinal cortex and hippocampus) during early stages, and then spreading to neocortical regions as the disease advances. This propagation pattern strongly correlates with worsening cognitive decline and ultimately drives clinical AD manifestations (Lewis and Dickson 2016). Notably, tau protein hyperphosphorylation is also tightly correlated with Aβ aggregation and oxidative stress. First, Aβ plaque deposition promotes cortical tauopathy through kinase activation (e.g., glycogen synthase kinase-3β [GSK3β]), inducing tau hyperphosphorylation and subsequent NFT formation that disrupts axonal transport (Huang et al. 2025). Then, oxidative stress further exacerbates tau pathology by detaching phosphorylated tau from microtubules, thereby impairing neuronal cytoarchitecture and transport mechanisms (Kumar and Singh 2015).

### Calcium Homeostasis Disruption Induced AD

**Fig. 4:**
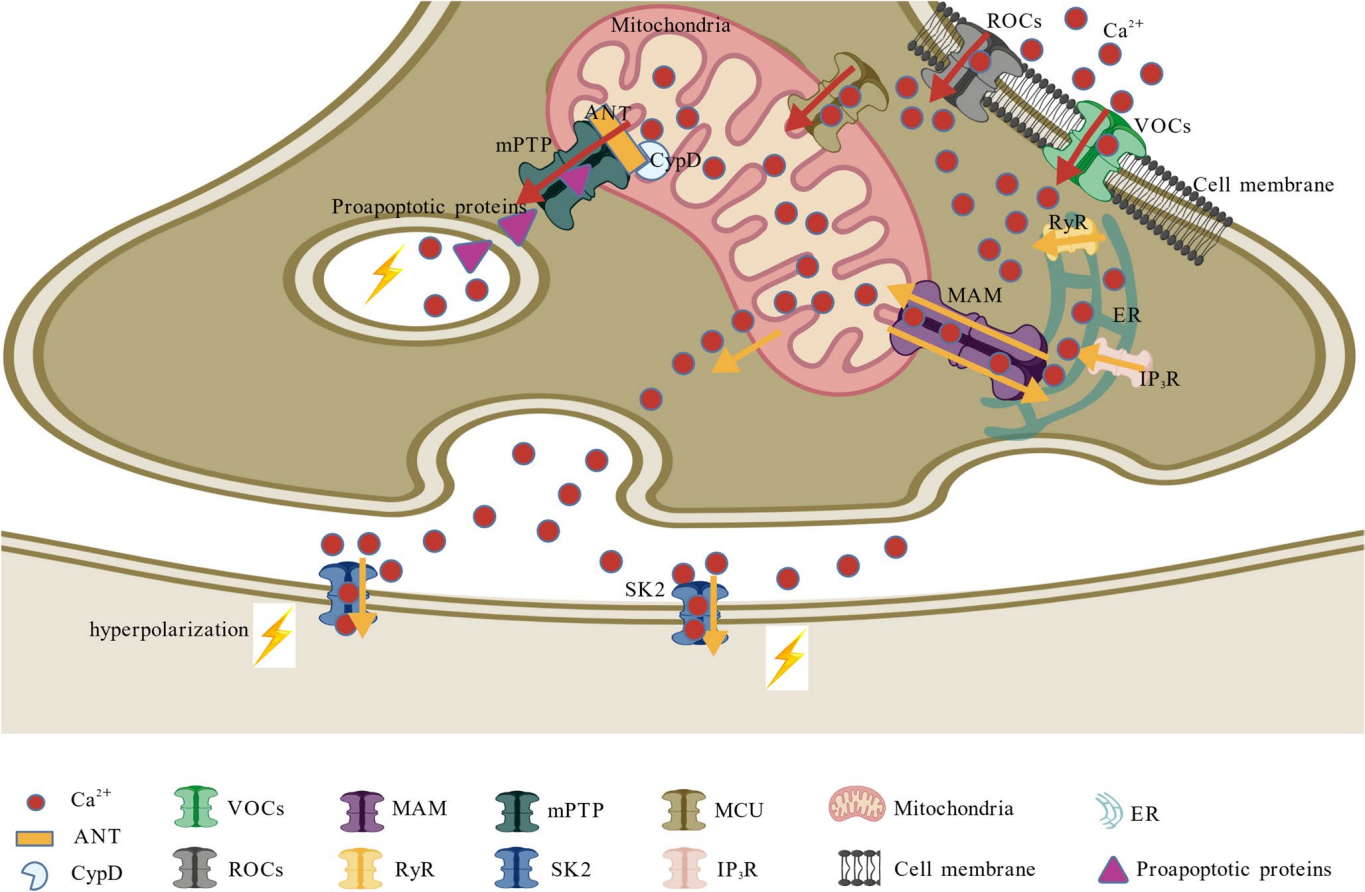
Ca²⁺ homeostasis disruption induced AD

Mounting evidence indicates that calcium (Ca²⁺) homeostasis disruption plays a pivotal role in AD pathogenesis, involving a cascade of pathological events originating at the cellular membrane and propagating to intracellular organelles (Fig. 4). Initial imbalances in Ca²⁺ regulation, driven by abnormal activity of voltage-gated channels (VOCs) and receptor-operated channels (ROCs) on the plasma membrane, lead to excessive extracellular Ca²⁺ (Ca²⁺_ext_) influx into the cytoplasm, thereby further compromising cytoplasmic Ca²⁺ (Ca²⁺_cyt_) homeostasis. This disruption triggers a vicious cycle whereby Ca²⁺_cyt_ is subsequently transported into mitochondria via the mitochondrial calcium uniporter (MCU), resulting in mitochondrial Ca²⁺ (Ca²⁺_mit_) overload. The consequent opening of the mitochondrial permeability transition pore (mPTP) releases pro-apoptotic factors that initiate neuronal apoptosis and neurodegeneration (Joshi et al. 2023). Meanwhile, presenilin (PS) mutations directly enhance Ca²⁺ release from endoplasmic reticulum (ER) stores through hyperactivation of ryanodine receptors (RyRs) and inositol triphosphate receptors (IP₃Rs), leading to excessive Ca²⁺_ext_ influx into the cytoplasm, thereby compromising Ca²⁺_cyt_ homeostasis. These ER-derived Ca²⁺ signals are further amplified at mitochondria-associated membranes (MAMs), creating a feedforward loop of calcium dysregulation. Notably, the pathological Ca²⁺ flux extends beyond intracellular compartments, diffusing to postsynaptic membranes where it overactivates Ca²-activated K⁺ channels (SK2), causing neuronal hyperpolarization and subsequent impairment of synaptic transmission. Concurrently, Ca²⁺-mediated destabilization of dendritic spine morphology undermines synaptic structural integrity, culminating in the characteristic memory deficits and cognitive decline that accelerate AD progression (Briggs et al. 2017). This comprehensive calcium hypothesis integrates dysfunction at the presynaptic, postsynaptic, and organelle levels within the pathophysiology of AD.

### Clearance of Aβ

AD is characterized by a complex pathophysiology that includes Aβ aggregation, cholinergic deficits, tau hyperphosphorylation, and disruptions in Ca²⁺ homeostasis, each of which has prompted the development of targeted therapeutic strategies. For example, nerve growth factor (NGF) signaling pathway restoration protects basal forebrain cholinergic neurons (Alam and Nixon 2023); resveratrol attenuates tau pathology by suppressing GSK3β activity and reducing NFTs in transgenic models (Wang and Pasinetti 2014); Ca²⁺ channel blockers alleviate the progression of Ca²⁺ dysregulation-driven AD (Korte et al. 2024); silymarin counters Aβ-induced oxidative neuronal damage (Wei et al. 2022); artemisinin B alleviates neuroinflammation by downregulating pro-inflammatory cytokines (Qiang et al. 2018). Although these interventions demonstrate efficacy, prioritizing the clearance of Aβ is crucial due to its central role in the pathogenesis of AD. As far as we know, Aβ is mainly cleared through four primary Aβ clearance pathways, as follows (Table 1), among which enzymatic degradation by plasmin offers distinct advantages. Its direct proteolytic action on Aβ ensures rapid and efficient clearance.

The first pathway is enzymatic degradation, which represents a primary mechanism for Aβ clearance. This direct proteolytic action ensures rapid and efficient Aβ clearance. Notably, the fibrinolytic system remains the only enzymatically validated degradation route (Angelucci et al. 2022). In vitro studies have revealed that plasmin selectively cleaves Aβ at arginine and lysine residues, generating non-toxic fragments including the middle-region peptide (LVFFAEDVGSNKG) and N-terminal segment (DAEFR), while exhibiting no neurotoxicity at therapeutic concentrations (Tucker et al. 2000). Besides plasmin, other fibrinolytic components are also involved in Aβ degradation. For example, exogenous administration of urokinase-type plasminogen activator (uPA) can rescue Aβ-mediated synaptic dysfunction (Diaz et al. 2020); the genetic deletion of plasminogen activator inhibitor-1 (PAI-1) enhances tissue-type plasminogen activator (tPA)-dependent clearance of Aβ plaques in AD models (Liu et al. 2011).

The second pathway is BBB transport, in which Aβ is cleared by being transported across the BBB into circulation. Therefore, some Aβ target-binding molecules have shown excellent exploratory promise. LRP-1 can directly bind to Aβ and transport it from brain tissue to vascular endothelial cells, which then exocytose it into the blood circulation (Akhter et al. 2018). P-gp can also directly bind to Aβ and, through conformational changes, move it from the brain parenchyma side to the arterial lumen side (Hartz et al. 2018). Complementing these endogenous pathways, certain monoclonal antibodies leverage immune-mediated clearance. Microglia are activated when Aducanumab (Aduhelm) attaches to the N-terminal portion of Aβ and interacts with Fc receptors on their surface via the antibody’s Fc segment. Through phagocytosis, activated microglia remove Aβ aggregates that have been identified by the antibody. The intracellular endosome/lysosome system then breaks down Aβ (Haddad et al. 2022). Lecanemab (BAN2401) is another monoclonal antibody targeting the N-terminal portion of Aβ for the treatment of AD (Cummings et al. 2023). Once bound to Aβ, BAN2401 can be recognized by receptors on the surface of macrophages and activate them. Then, the ingested antibody-Aβ aggregate complexes are transported by macrophages into the cells’ lysosomes, which contain a variety of hydrolases that can degrade Aβ aggregates into smaller molecules and remove Aβ (Chen et al. 2023). Most recently, Donanemab was approved by the FDA for the treatment of AD; it is directed at the clearance of a post-translationally modified form of Aβ (pE3-Aβ) (Mintun et al. 2021).

The third pathway is the glymphatic system (GS), comprising meningeal lymphatic vessels and the perivascular glial-lymphatic network. Central to this system is aquaporin-4 (AQP4)-mediated directional fluid flow, which channels Aβ-laden interstitial fluid along perivenous spaces for eventual drainage into meningeal lymphatic capillaries or peripheral circulation. Disrupted AQP4 polarization in AD models significantly compromises clearance efficiency. Meanwhile, this impairment creates a vicious cycle: microinfarctions damage the neurovascular unit, obstruct glymphatic flow, and exacerbate metabolic waste deposition at lesion sites (Ben-Nejma et al. 2023). Moreover, such vascular insults trigger concurrent tau phosphorylation and Aβ aggregation (Lin et al. 2022). Notably, human studies have confirmed that loss of polarized AQP4 localization correlates with accelerated AD pathology (Simon et al. 2022), suggesting that glymphatic enhancement after microinfarction could represent a novel therapeutic strategy for interrupting this degenerative cascade.

The fourth pathway is peripheral clearance, which mainly comprises immune cell-mediated clearance and renal-mediated clearance. In the former system, PSK potently activates Toll-like receptor 2 (TLR2) on monocytes, enhancing their capacity to detect, internalize, and degrade Aβ through lysosomal pathways, while simultaneously inhibiting peripheral Aβ re-entry into the brain. This dual action reduces cerebral Aβ burden, attenuates neuroinflammation, and ultimately ameliorates cognitive deficits in AD models, demonstrating the therapeutic potential of modulating peripheral monocyte function (Chen et al. 2022). Meanwhile, the activation of macrophages in a peroxisome proliferator-activated receptor gamma (PPARγ)-dependent manner via erythropoietin (EPO) enhances peripheral Aβ catabolism (Xu et al. 2022). In the latter system, renal tubular epithelial cells in AD models can actively clear circulating Aβ via megalin-mediated endocytosis, followed by either lysosomal degradation (facilitated by renal Aβ-degrading enzymes such as neprilysin) or direct urinary excretion. Moreover, acute renal artery ligation experiments further established a direct correlation between impaired renal function and rapid elevation of Aβ levels in both blood and brain interstitial fluid (ISF), underscoring the kidney’s role in maintaining systemic Aβ homeostasis and its indirect influence on cerebral Aβ dynamics (Tian et al. 2021). These findings collectively position peripheral clearance pathways as promising therapeutic targets for AD intervention.

Among these aforementioned clearance pathways, the enzymatic degradation pathway stands out as the most therapeutically significant due to its high efficiency and specificity. Notably, fibrinolytic enzyme (plasmin) remains the only demonstrated agent able to enzymatically degrade Aβ aggregates directly and is also strongly implicated in AD pathogenesis (Angelucci et al. 2022). Therefore, enhancing endogenous plasmin activity or administering exogenous plasmin might represent a promising therapeutic strategy for AD, which could effectively reduce cerebral Aβ burden, mitigate its neurotoxic effects, and ultimately attenuate AD progression.

**Table 1:** Agents and approaches applied in Aβ clearance

Category	Agents	Mechanism	Effects	References
Clinical drug	Aducanumab	It specifically recognizes the conformational epitope of Aβ spanning amino acid residues 3–7 at the N-terminus, binds fibrillar Aβ aggregates with high affinity, dissociates them into non-toxic monomers, and thus indirectly mitigates Aβ-induced neurotoxicity.	Reduce Aβ plaques in the brain, restore neural function.	(Haddad et al. 2022)
	Donanemab	It binds N-terminal pyroglutaminated Aβ (position 2) to rapidly and fully clear amyloid deposits.	It significantly attenuated the deterioration of patients’ cognition and ADL.	(Mintun et al. 2021)
	Lecanemab	Targeting Aβ protofibrils, it boosts microglial phagocytosis to degrade them and reduce Aβ toxicity.	Reduce toxic Aβ aggregates in the brain, restore cognitive function.	(Chen et al. 2023)
	Bapineuzumab	It targets Aβ’s specific epitopes, binds all brain Aβ states, and mitigates its pathological buildup.	Did not show significant clinical benefits in patients with AD.	(Ivanoiu et al. 2016)
	Gantenerumab	Specific binds to Aβ, activates immunophagic processes, and facilitates extra-cerebral transport.	Significantly reduce cerebral Aβ deposition in patients with early-stage AD.	(Silvestri et al. 2024)
	IVIg/FUS	Anti-Aβ antibodies in IVIg target aggregated brain Aβ; FUS-delivered IVIg interacts with hippocampal Aβ plaques to disrupt fibrils, prevent plaque progression, and reduce neurotoxicity.	Indirectly reduces Aβ production in the brain.	(Dubey et al. 2020)
	GV-971	It protects the BBB transport-mediated Aβ clearance and indirectly enhances extra-cerebral Aβ transport efficiency.	Reduce the accumulation of Aβ in the brain.	(Yang et al. 2024)
	Aducanumab and SUS	Aducanumab directly binds and clears Aβ, while SUS indirectly promotes Aβ clearance by opening the BBB and activating microglia; their combination synergistically boosts Aβ clearance via enhanced antibody brain delivery.	Improving the efficiency of Aβ clearance.	(Leinenga et al. 2021)
Preclinical agent	LRP-1	It binds to Aβ, transports Aβ from brain tissue to the blood, and reduces Aβ deposition in the brain.	Reduce Aβ deposition in the brain.	(Akhter et al. 2018)
	P-gp	It binds to Aβ and moves Aβ from the brain parenchyma side to the arterial lumen side through conformational changes.	Reduce Aβ deposition in the brain.	(Hartz et al. 2018)
	Megalin	Megalin-bound Aβ undergoes receptor-mediated endocytosis to form complex-containing endosomes, which are sorted to lysosomes for Aβ degradation into non-toxic short peptides by cathepsins B, D, and other enzymes.	Reduce the aggregation of Aβ.	(Tian et al. 2021)
	LRP1/apoE	Pericyte-internalized Aβ-LRP1-apoE complexes undergo lysosomal degradation by proteases (e.g., cathepsins) into non-toxic short peptides.	Clear Aβ aggregates.	(Ma et al. 2024)
	plasmin	Targeting Aβ’s hydrophobic sequence (soluble Aβ₄₀/Aβ₄₂ monomers/oligomers), it cleaves key Aβ₄₂ peptide bonds to generate non-fibrillogenic, non-toxic fragments (Aβ₁₋₁₀, Aβ₁₋₂₀) that are peripherally cleared via the glymphatic system or BBB.	Potent degradation of Aβ directly.	(Angelucci et al. 2022)
	Neprilysin (NEP)	Targets Aβ’s C-terminal hydrophobic sequence, cleaves key Aβ₄₂ peptide bonds to generate non-aggregating fragments (Aβ₁₋₁₀/Aβ₁₋₂₀) for peripheral excretion.	Reduce the level of Aβ in the brain.	(Tian et al. 2021)
	tPA	tPA activates plasminogen into plasmin.	Indirectly accelerates the degradation of Aβ.	(Melchor et al. 2003)
	PAI-1	Inhibits tPA/uPA.	Indirectly accelerates the degradation of Aβ.	(Liu et al. 2008)
	AQP4	Mediates the flow of water molecules from the PVS into the brain parenchyma, which in turn drives the movement of Aβ from the brain parenchyma to the PVS.	Reduce the accumulation of Aβ in the brain	(Simon et al. 2022)
	PSK	PSK enhances the recognition, uptake, and lysosomal degradation of Aβ by activating TLR2 receptors, while reducing the transport of peripheral Aβ into the brain.	Reduce Aβ deposition in the brain, alleviating neuroinflammation, and neuronal damage.	(Chen et al. 2022)
	Somatostatin	Regulates Aβ catabolism by enhancing NEP-catalyzed proteolytic degradation.	Attenuated Aβ deposition indirectly.	(Watamura et al. 2022)
	Voluntary wheel running	Accelerates the clearance function of the brain’s glymphatic system and regulates the expression and localization of AQP4 in astrocytes.	Indirectly reduces the accumulation of Aβ.	(He et al. 2017)
	CLPFFD	Precisely delivers gold nanoparticles with photothermal properties to Aβ targets.	Inhibit Aβ aggregation, disassemble pre-formed Aβ fibrils”.	(Ruff et al. 2018)

### The Dual Role of the Fibrinolytic System in AD

The fibrinolytic system, known for its ability to break down fibrin, has been primarily associated with the removal of blood clots. It is widely acknowledged that the fibrinolytic system is involved in many physiological and pathophysiological processes. Accumulating evidence has demonstrated a neuroprotective and anti-AD potential of the fibrinolytic system. Pradoxically, more recent research also indicated a pro-AD effect of this system, highlighting its dualistic role in AD pathogenesis. This paradigm shift establishes this system as a potential therapeutic target for AD, albeit requiring precise modulation to balance its beneficial and detrimental effects (Table 2).

**Table 2:** The dual role of the firbinolytic system in AD

Agent	Effect	Pathway	References
plasmin	Anti-AD	It cleaves Aβ directly, which in turn reduces Aβ plaque deposition.	(Angelucci et al. 2022)
		Clears microthrombi and fibrin deposits from vascular walls, maintaining cerebral blood perfusion and microcirculatory patency.	(Akhter et al. 2018)
		Prevents Aβ from binding to fibrinogen to form complexes, thereby inhibiting the occurrence of neuroinflammation.	(Singh et al. 2018)
		Converts Pro-BDNF to mBDNF which enhances synaptic plasticity and sustains neuronal survival.	(Laske and Eschweiler 2006)
	Pro-AD	Activates macrophages which breaches the BBB, infiltrates the brain parenchyma, and secretes proinflammatory cytokines.	(Baker et al. 2018)
		Activates microglia and astrocytes which release proinflammatory cytokines.	(Baker et al. 2018)
Plasmin regulators	Anti-/pro-AD	Regulate the activity of plasmin.	(Melchor et al. 2003)
tPA	Pro-AD	Binds to NMDAR, G proteins, and PKC, which in turn induces tau hyperphosphorylation through the activation of the Erk1/2-GSK3 pathway.	(Medina et al. 2005)

### Composition and Distribution of Fibrinolytic System

**Fig. 5:**
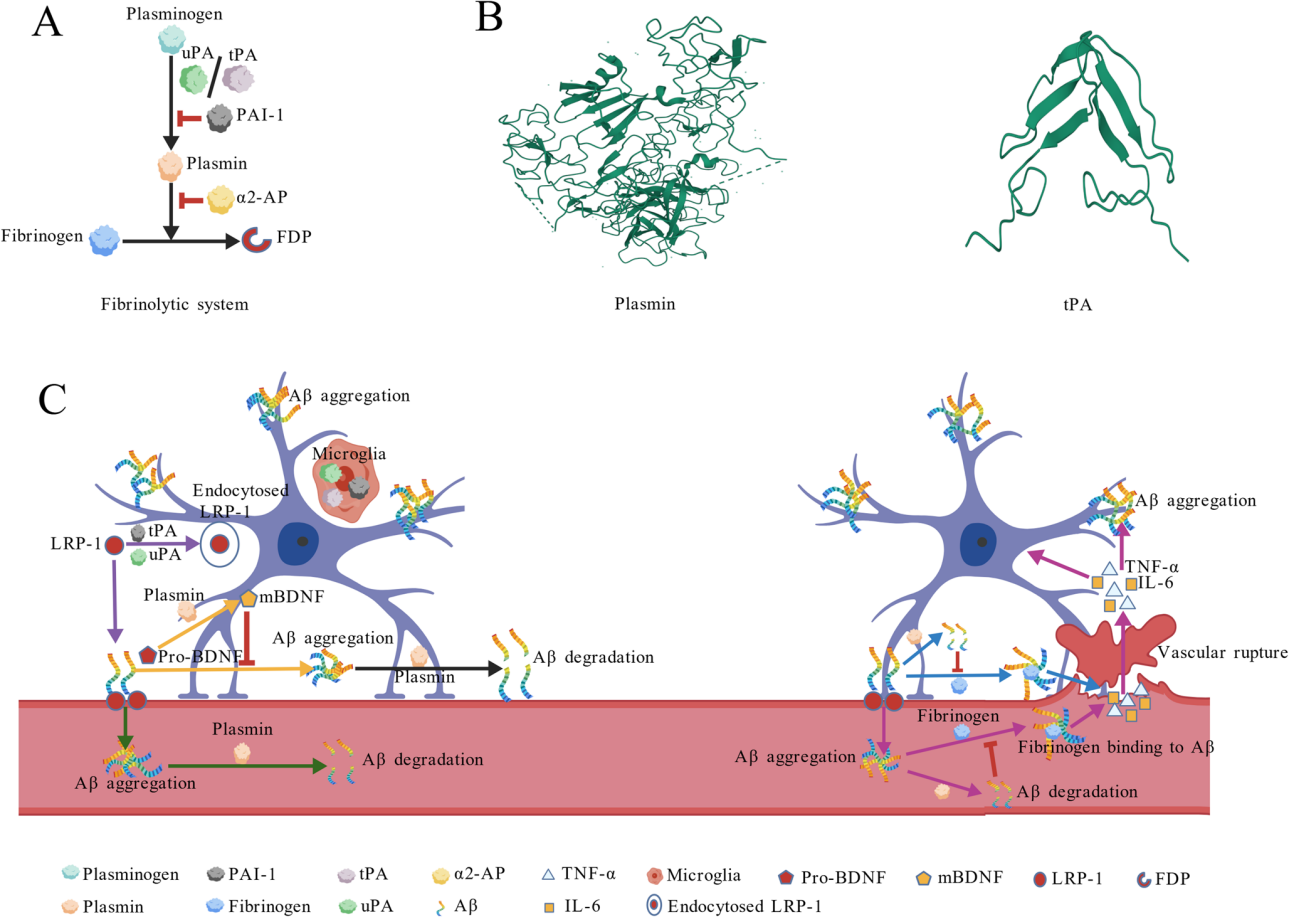
Anti-AD effects of fibrinolytic system. **A** Schematic map of the conventional fibrinolytic system; **B** Representative structures of plasmin and tPA; **C** Anti-AD pathways of fibrinolytic system: degrade Aβ directly (indicated with black arrow), improve the microenvironment of CNS (indicated with yellow arrows), and, improve the microenvironment of cerebrovascular (indicated with purple and red arrows)

Conventionally, the fibrinolytic system consists of plasminogen, plasminogen activators (PAs) such as tPA and uPA, and plasminogen activator inhibitors (PAIs) such as PAI-1 and α2-antifibrinolytic (α2-AP) (Medcalf and Keragala 2021; Keragala and Medcalf 2021). This system is a cascade of protein activation events, which starts with the conversion of plasminogen to a catalytically active enzyme (plasmin) by PAs, followed by fibrin degradation by plasmin. Meanwhile, the activities of PAs and plasmin are inhibited by PAI-1 and α2-AP, respectively (Fig. 5A and **B**) (Mutch and Medcalf 2023; Seillier et al. 2022; Merino et al. 2017; Tetsumei et al. 2019). Therefore, increasing attention has been focused on fibrinolytic agents that possess both direct fibrinolytic activity and plasminogen-activation activity (Mintun et al. 2021). Notably, snFPITE was reported first by our team, which possesses a novel plasminogen activation mechanism and fibrin degradation mechanism, and shows great promise for the treatment of thrombosis and other related protein deposition disorders (Tang et al. 2023, 2025; Lin et al. 2023; Kang et al. 2024).

Normally, circulating plasminogen and PAI-1 are mainly produced in the liver, and PAs are produced in vascular endothelial cells (Shinoda-Ito et al. 2023; Zheng et al. 2018, 2020). However, these circulating fibrinolytic agents are virtually unable to cross the BBB and enter the central nervous system (CNS) under normal physiological conditions. Therefore, an intrinsic fibrinolytic system is present in the CNS via endogenous synthesis (Tsirka et al. 1997). Neurons predominantly produce tPA, which is stored in synaptic vesicles for activity-dependent release (Jeanneret et al. 2016). Additionally, astrocytes and microglia contribute to CNS fibrinolysis by synthesizing tPA and PAI-1 (Wilhelm et al. 2018; Blackmon et al. 2025).

### The Anti-AD Effects of the Fibrinolytic System

The pathophysiology of AD is characterized by a multifaceted cascade involving Aβ aggregation as the central pathological driver. Interestingly, fibrinolytic enzyme (plasmin) remains the only demonstrated agent capable of directly enzymatically degrading Aβ aggregates directly. Besides this direct Aβ degradation pathway, the improvement of the CNS and cerebrovascular microenvironment pathways is also involved in the anti-AD effects of the fibrinolytic system (Fig. 5C).

### Degrade Aβ Aggregates Directly

The inverse correlation between plasmin activity and Aβ deposition is well established, with reduced plasmin levels in AD brains exacerbating the accumulation of both soluble and aggregated Aβ species (Ali et al. 2024). Further clinical data have also confirmed this phenomenon (Guo et al. 2023). Although plasmin remains the only active fibrinolytic agent in the entire fibrinolytic system, free plasmin is rapidly quenched quickly in circulation by inhibitors, which limits its application in anti-AD studies. However, Yang et al. engineered µPlm variants that maintain proteolytic activity while resisting inhibition, presenting a promising therapeutic avenue (Yang et al. 2020). Another approach adopted to overcome these physiological inhibitors is to replace plasmin with other fibrinolytic components, as its activity/level is tightly regulated by other components. such as plasminogen, PAs, and inhibitors. A comparative study showed that while wild-type mice rapidly eliminate hippocampally injected Aβ, plasminogen-deficient mice exhibit markedly delayed clearance (Melchor et al. 2003). Moreover, in AD mouse models and clinical patients, exogenous plasminogen supplementation (i.v.) can cross the BBB, concurrently degrading Aβ and tau while restoring cholinergic function and memory performance (Guo et al. 2023). PAs such as tPA and inhibitors such as PAI-1 have also emerged as significant contributors to AD pathogenesis, with elevated plasma PAI-1 levels serving as a potential biomarker for early AD detection (Oh et al. 2014; Eruysal et al. 2022). In tPA-deficient mouse models, markedly delayed clearance of hippocampal-injected Aβ was observed when compared with the wild-type group (Melchor et al. 2003). Mechanistically, increased tPA activity or decreased PAI-1 activity promotes both tPA-mediated plasmin generation and subsequent Aβ degradation, thereby alleviating AD pathology. By using specific PAI-1 inhibitors, Steven Jacobsen et al. demonstrated that PAI-1 inhibitors augment the activity of tPA and plasmin in the hippocampus, significantly lower plasma and brain Aβ levels, restore long-term potentiation deficits in hippocampal slices from transgenic Aβ-producing mice, and reverse cognitive deficits in these mice (Jacobsen et al. 2008). Gene knockout mouse models further demonstrated the pivotal role of tPA and PAI-1 in Aβ degradation. For example, the genetic ablation of PAI-1 reduces cerebral Aβ burden in AD mouse models by enhancing tPA-dependent fibrinolysis of Aβ plaques (Liu et al. 2011); the genetic ablation of PAI-1 in APP/PS1 transgenic mice enhances tPA-mediated plasmin activation, leading to a significant reduction in cerebral Aβ burden and attenuation of AD progression (Liu et al. 2011); tPA knockout mice display slowed Aβ clearance following intracerebral injection, whereas PAI-1 knockout mice exhibit accelerated clearance (Seeds and Fabbro 2009).

### Improve the CNS Microenvironment

The specific microenvironment of the CNS is vital for proper neuronal function. The reparative or destructive outcome depends on the molecular context of the CNS microenvironment. With regard to the microenvironment of the CNS, it is important to mBDNF, a key neurotrophin. There are different isoforms of BDNF, such as mBDNF and Pro-BDNF. BDNF is a 13-kDa protein that is synthesized de novo in neurons and released as both mBDNF and Pro-BDNF. Pro-BDNF is taken up and released by astrocytes. Subsequently, mBDNF attenuates Aβ deposition, promotes long-term potentiation, and exerts anti-apoptotic effects (Budni et al. 2015). Pro-BDNF accelerates Aβ deposition, induces apoptosis, and promotes long-term depression (Chen et al. 2017). Recent studies have demonstrated that the fibrinolytic system also critically regulates neurotrophic signaling. PAs have been reported to enhance the conversion of Pro-BDNF into mBDNF through plasmin activity (Laske and Eschweiler 2006). Another research team confirmed that PAI-1-mediated inhibition of PA activity indirectly reduces mBDNF synthesis, thereby impairing synaptic plasticity and neuronal survival (Angelucci et al. 2019). Therefore, based on the observation that the decrease in plasmin levels caused by PAI-1 may negatively impact mBDNF production and promote disease progression, the ratio of PAI-1 to mBDNF may serve as a promising biomarker for AD diagnosis (Angelucci et al. 2023). Moreover, the tPA-plasmin system regulates synaptic plasticity through the precise proteolytic processing of Pro-BDNF at the Arg125-Val126 cleavage site (Gray and Ellis 2008).

As a core pathological hallmark of neurodegenerative diseases, oxidative stress compromises the BDNF system through multiple mechanistic layers: on one hand, ROS directly perturb BDNF synthesis-associated signaling cascades in neurons and abrogate tPA/plasmin-mediated Pro-BDNF maturation; on the other hand, oxidative stress-elicited inflammatory cytokines (e.g., IL-6, TNF-α) upregulate the expression of PAI-1, which forms a stable complex with tPA to abrogate its enzymatic activity, ultimately resulting in inadequate biosynthesis of mBDNF (Hoirisch-Clapauch 2022). Therefore, it is reasonable that the dysfunction of NAD (P)H quinone dehydrogenase 1 (NQO1) contributes to exacerbated oxidative stress in the CNS, whereas plasmin may indirectly modulate redox balance by regulating abnormalities in neurotransmitter release or clearance (e.g., under conditions of impaired NQO1 activity) (Yuhan et al. 2024).

### Improve the Cerebrovascular Microenvironment

Because PAI-1 is a pivotal cofactor of uPA-uPA receptor (uPAR)-mediated low-density LRP1 endocytosis, inhibitors of PAI-1 can significantly reduce LRP1 endocytosis and enhancing the availability of LRP1 on the BBB membrane. Higher LRP1 levels enable in current literaturegreater Aβ efflux from the brain to the bloodstream, promoting active Aβ excretion (Akhter et al. 2018).

Additionally, it has been demonstrated that Aβ exhibits a high binding affinity for fibrinogen, resulting in the formation of structurally abnormal fibrin clots that are resistant to fibrinolysis. These pathological clots disturb the physiological balance between coagulation and fibrinolysis, leading to prolonged thrombolysis and circulatory impairments associated with vascular occlusion. Scanning electron microscopy reveals that Aβ-fibrinogen complexes induce malformed fibrin networks characterized by thin, disorganized filaments and dense clot aggregates, which alter intravascular coagulation architecture and exacerbate cerebrovascular damage. Critically, this vascular pathology creates a vicious cycle: Aβ-fibrinogen-mediated microvascular occlusion and BBB compromise facilitate the influx of peripheral inflammatory factors, triggering sustained neuroinflammation through glial activation that ultimately drives neuronal injury and cognitive decline (Singh et al. 2018). This phenomenon is further substantiated by independent studies confirming that fibrinogen accumulation in AD brains synergizes with Aβ to accelerate disease progression (Casquero-Veiga et al. 2025). Therefore, plasmin emerges as a key modulator of this pathway through fibrinogen proteolysis, effectively reducing Aβ-fibrinogen interactions. However, the potential binding capacity of fibrin or other fibrin degradation products (FDPs) to Aβ remains unexplored in the current literature, representing a significant gap in our understanding of the complete fibrinolytic-Aβ interaction.

### The Pro-AD Effects of the Fibrinolytic System

**Fig. 6:**
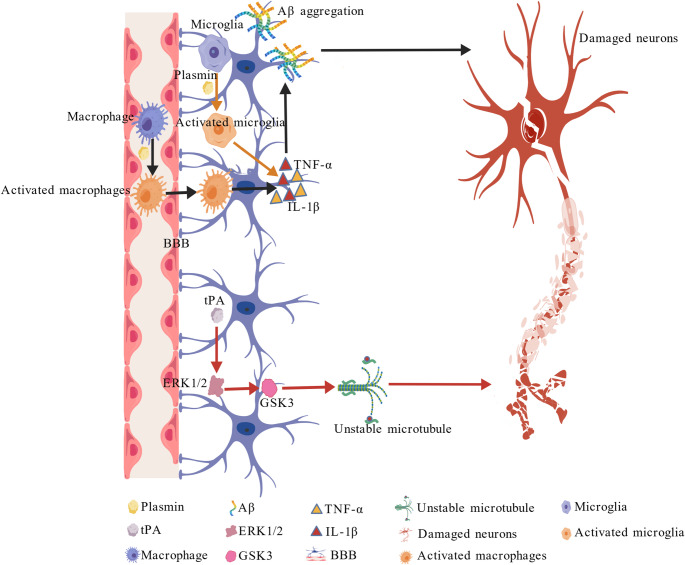
Pro-AD effects of the fibrinolytic system. Plasmin-mediated neuroinflammation through glial activation and macrophage infiltration (indicated with black and brown arrows) and tPA-mediated tau hyperphosphorylation via the ERK1/2-GSK3 axis (indicated with red arrows)

Although the potent anti-AD effects of the fibrinolytic system were widely acknowledged, emerging evidence indicates that this system may also exhibit pro-AD effects, which deserve more attention in the future. There are mainly two pathways involved in its pro-AD effects: (1) Plasmin-mediated neuroinflammation through glial activation and macrophage infiltration; (2) tPA-mediated tau hyperphosphorylation via the extracellular signal-regulated kinase 1/2 (ERK1/2)-GSK3 axis (Fig. 6).

### Plasmin-Mediated Neuroinflammation

As early as 2018, Barker et al. noted that depletion of plasminogen in the plasma of an AD mouse model through antisense oligonucleotide technology dramatically improved AD pathology and decreased glial cell activation in the brain, whereas an increase in plasmin activity through α2AP antisense oligonucleotide treatment exacerbated the brain’s immune response and plaque deposition (Baker et al. 2018). These results suggest a crucial role for peripheral plasmin in mediating neuroimmune cell activation and AD progression, and could provide a link to systemic inflammatory risk factors known to be associated with AD development. Meanwhile, plasmin facilitates the infiltration of perivascular macrophages (PVMs) into the CNS and promotes PVM secretion of pro-inflammatory factors, which activate microglia and astrocytes, thereby amplifying neuroinflammatory responses. On the other hand, lipopolysaccharide (LPS)-induced peripheral inflammation triggers the conversion of plasminogen to plasmin. Therefore, plasminogen can mediate the transmission of peripheral immune signals to the central nervous system, exacerbating neuroinflammation through glia activation (Baker et al. 2019). These cascading events collectively establish a chronic neuroinflammatory microenvironment. Moreover, in AD mouse models, peripheral plasminogen can cross the BBB to activate microglia and astrocytes in the brain, promoting the release of pro-inflammatory cytokines and establishing a chronic neuroinflammatory milieu. Further study showed that plasminogen may activate astrocytes to express pro-inflammatory cytokines through the concerted action of at least three distinct fibrinolytic protease receptors. This pathway is dependent on uPA to activate plasminogen; uPA is endogenously expressed by cultured astrocytes and may also be provided by other cells in the astrocytic microenvironment of the CNS (Pontecorvi et al. 2019).

Aβ₄₂ can bind to fibrinogen to form a corresponding complex, which exhibits antiplasmin activity and induces synaptic toxicity, neuroinflammation, and BBB disruption. These complexes activate microglia to promote proinflammatory cytokine release while inhibiting plasmin’s normal degradative function (Simoes-Pires et al. 2025). In severe tissue injury (e.g., burns), plasmin activation acts as a key pathological driver of systemic inflammatory response syndrome (SIRS) and cytokine storms by amplifying inflammatory signaling (e.g., NF-κB pathway) in distant damaged tissues (Gibson et al. 2021).

### tPA-Mediated Tau Hyperphosphorylation

Besides the fibrinolysis-dependent pro-inflammatory effects mentioned above, a fibrinolytic-independent pro-AD pathway may also exist. Recent studies demonstrate that tPA induces neuronal death through an ERK1/2-dependent pathway that is independent of its catalytic activity. In the CNS, tPA activates ERK1/2, which further stimulates GSK3 via de novo protein synthesis. This GSK3-mediated hyperphosphorylation of tau at the tau-1 (Ser198/Ser202/Thr205) and AT180 (Thr231) epitopes destabilizes microtubules, contributing to AD pathogenesis. Immunohistochemical analyses confirm the colocalization of phosphorylated tau and activated ERK1/2 in AD brains. Notably, tPA activity shows a significant age-related decline, potentially exacerbating tau pathology (Medina et al. 2005). tPA directly modulates synaptic plasticity via binding to synaptic receptors, including NMDAR, LRP1, Annexin II, and EGFR (Varangot et al. 2023). Specifically, the interaction between tPA and NMDAR enhances calcium influx, activates calcium-dependent kinases (e.g., GSK3β), and thereby promotes the hyperphosphorylation of tau protein at the Ser396, Thr181, and Thr231 sites (Tang et al. 2022; Choudhury et al. 2025). Additionally, tPA can cleave brain-derived Pro-BDNF into mBDNF, thus regulating TrkB receptor signaling. Dysregulation of this process may induce an imbalance between neuroprotective and neurotoxic effects, exacerbating tau pathology (Varangot et al. 2023).

## Conclusions

AD, the most prevalent neurodegenerative disorder among the elderly, is characterized by progressive dementia that significantly impacts quality of life. Over a century of research has evolved from clinical observations to a molecular-level understanding of its pathogenesis, which progresses through three stages: early pathological initiation, synaptic dysfunction, and advanced neuronal death with structural brain damage (dementia phase). The disease’s complex pathophysiology centers on Aβ aggregation as the primary driver, which interacts with cholinergic deficiency, tau hyperphosphorylation, and calcium dysregulation. These processes are exacerbated by oxidative stress and neuroinflammation, which not only accelerate Aβ deposition but also promote tau pathology, ultimately triggering synaptic failure and clinical onset. Current therapeutic strategies, including dietary polyphenols and resveratrol, show partial efficacy, as they primarily address symptoms rather than fundamentally targeting Aβ aggregates. This limitation has spurred interest in Aβ clearance, which occurs through enzymatic degradation, BBB transport, GS, and peripheral clearance. Among these, enzymatic degradation by the fibrinolytic system is particularly promising due to its specificity and efficiency.

However, the fibrinolytic system exhibits a dual role in AD, mitigating AD pathology by directly cleaving Aβ while paradoxically exacerbating AD progression through peripheral-to-central immune signaling-mediated neuroinflammation (Baker et al. 2018; Angelucci et al. 2022). Although the specific turning point of such duality remains unknown, emerging evidence suggests that this double-edged sword effect is context-dependent (Angelucci et al. 2019). Once overactivated, the system stimulates perivascular macrophages to migrate into brain tissue and release pro-inflammatory factors, thereby accelerating Aβ aggregation. Therefore, it is urgent to identify whether the fibrinolytic domain is identical to its inflammation-activating domain. Only when its active domains is clarified can more selective approaches be adopted to retain its anti-AD effects while avoiding its pro-AD effects.

In terms of therapeutic efficacy against AD, it is feasible to achieve clinically interventions by targeting specific domains or pathways, with the key lying in enhancing specificity and local control. A multifunctional liposome delivery system, KLVFF@LIP-CeO₂ was reported for the nasal co-delivery of the Aβ-targeting peptide KLVFF and ROS-responsive cerium oxide (CeO₂). Cellular models results showed that this formulation inhibited Aβ aggregation, scavenged ROS, and antagonized cell apoptosis. Further experiments in APPswe/PS1 transgenic mice experiments also confirmed that it efficiently accumulated in the brain, alleviated Aβ deposition and oxidative stress, and ameliorated cognitive impairment, exhibiting promising clinical potential for the synergistic treatment of AD (Shan et al. 2024). Recently, we intend to develop a novel multifunctional liposome delivery system, KLVFF@LIP-snFPITE, for the nasal co-delivery of the Aβ-targeting peptide KLVFF and snFPITE. targeted Aβ aggregation inhibition and degradation, thereby providing a safe and efficient novel strategy for AD therapy.

Regarding the aforementioned AD-promoting phenomenon, we proposed two potential strategies here: one is to inhibit macrophage activation, thereby preventing the release of inflammatory cytokines and preserving the physiological functions of plasmin in the bloodstream; the other is to block macrophage migration into brain tissue, which would attenuate the levels of inflammatory cytokines in the brain, further reduce Aβ deposition, and ultimately alleviate the pathological symptoms of AD. Meanwhile, fibrinogen-Aβ interactions play a pivotal role in AD pathogenesis (Singh et al. 2018). In AD patients, fibrinogen infiltrates the brain parenchyma through a compromised BBB, while Aβ efflux to the systemic circulation remains at trace levels. Once fibrinogen interacts with Aβ, this complex triggers vascular damage and inflammatory cascades, further disrupting BBB integrity and facilitating the invasion of neurotoxic factors. In this situation, limiting hepatic fibrinogen synthesis might represent a promising approach for mitigating such pro-AD effects induced by fibrinogen-Aβ interactions. Other strategies are also worth trying, such as targeted interventions—specifically, preventing this complex formation by developing monoclonal antibodies or small-molecule inhibitors against specific Aβ-binding domains.In conclusion, it is well established that the fibrinolytic system exerts double-edged sword effects in AD. This dual nature should serve as the foundation for future novel strategies for AD therapy.

## Supplementary Information

Below is the link to the electronic supplementary material.

Supplementary material 1 (7Z 5321.5 kb)

Supplementary material 2 (RAR 5929.1 kb)

## Data Availability

No datasets were generated or analysed during the current study.
